# Community Views on ‘What I Want ‘Before I Die’

**DOI:** 10.3390/bs8120111

**Published:** 2018-11-30

**Authors:** Deb Rawlings, Lauren Miller-Lewis, Jennifer Tieman

**Affiliations:** Palliative Care, College of Nursing and Health Sciences, Flinders University, Adelaide SA 5000, Australia; Lauren.miller@flinders.edu.au (L.M.-L.); jennifer.tieman@flinders.edu.au (J.T.)

**Keywords:** death and dying, MOOC, community conversations

## Abstract

Few previous studies have formally examined people’s wishes regarding what they want to do before they die. This study aimed to describe responses to an activity within a Massive Open Online Course (MOOC) where people considered what was important when faced with their own mortality. We asked participants to complete the following: “Before I Die, I want to…”. The content of participants’ responses (*n* = 633) was analysed qualitatively with a coding schema developed and then applied. All authors independently coded the first 100 “Before I Die” statements, followed by a second round of coding where themes were verified and confirmed. Following this, two independent raters coded all 633 responses, obtaining 95.24% agreement (Cohen’s Kappa = 0.789, *p* < 0.0005). Twelve themes emerged from the data: family; do an activity; personal aspiration; live life fully, happiness; love; the greater good; peace; legacy; gratitude; religion; and health. Responses could also be distinguished as being inward-facing (about the self), and outward-facing (about others). Reflecting on what is important and on what a person wishes to achieve or address before they die can be seen as a companion process to advance care planning which addresses what an individual wants to plan to manage their actual death.

## 1. Introduction

A local art project was developed in New Orleans by Candy Chang following the death of someone she loved, in the realisation that we, as a society, have stopped talking about death and dying [1]. This participatory public art project provided a space where people could start a conversation, writing their wishes and aspirations (“Before I Die I want to…”) on a public chalkboard [1]. This endeavour has since become a global social change phenomenon with over 4000 “*Before I Die”* walls, developed in over 75 countries and in 36 languages (https://beforeidieproject.com/).

Bringing death back into the open and engaging communities in death and dying conversations has been seen in the emergence of death cafés [2] and in the compassionate community’s movement [3], viewing death and dying and community engagement through a public health lens expressed in art, photography, and social media [4]. Similar to the “Before I Die” philosophy is that of the “bucket list”, made popular in recent years often with a “you only live once” [5] approach to life. Bucket lists often include very action-oriented aspirations, things not previously done and yet to be ticked off. Bucket lists can be a wish list of the unachieved, a recognition of our own mortality, or a reflection of personal values [6].

Few previous studies have examined people’s wishes regarding what they want to do before they die. Periyakoil and colleagues [6] administered an anonymous online public survey to 3056 participants across the United States asking them to list things on their bucket list, defined as “things that one has not done before but wants to do before dying” (p. 2). These authors identified six primary themes: travel, accomplish a personal goal, achieve specific life milestones, spend quality time with friends and family, achieve financial stability, and do a daring activity. In a sample of 391 messages on an open media wishboard where people wrote responses to the question “for the next year I want…”. Ferreria and colleagues [7] compared their findings with those of Candy Chang’s work. They found similar patterns although highlighted that responses may be contextual to the space/locality in which they are offered [7]. Chang’s book [1] descriptively summarised results from what is considered important to over 100,000 people highlighting “Wellbeing”, “Love”, “Travel”, and “Helping family” as the most important. Arguably, a more in-depth systematic empirical qualitative analysis examining responses to the “Before I Die” activity is warranted and could contribute further to knowledge about people’s wishes in this arena.

In 2016 we offered for the first time, a Massive Open Online Course (MOOC) called Dying2Learn [8]. The MOOC was developed within the “cMOOC” constructivist approach aiming for a peer-to-peer community-driven experience focussing on fostering connection, rapport and openly discussing death and dying [9]. Participants conversed on all things to do with death and dying, its meaning for them, their patients (if they were health professionals), their families, their colleagues and the broader community. The MOOC was offered again in 2017 and in each MOOC, we have included a virtual “Before I Die” wall within the learning platform (with permission and acknowledgement). The aim of this study with a community sample, was to describe what things people consider important when faced with their own mortality through an online activity designed to bring death to the forefront of their mind by asking “Before I die, I want to…”.

## 2. Methodology

Ethical approval related to the project was granted by Flinders University Social and Behavioural Research Ethics Committee (Project 7247).

### 2.1. Participants

The “Before I Die” activity was completed in the final week of the 2016 and 2017 Dying2learn courses. In 2016, 1156 people enrolled in the course (posting 9872 comments) with 219 participating in the “Before I Die” activity. The 2017 MOOC saw 1960 enrolees (posting 12,042 comments), with 414 participating in the “Before I Die” activity. The “Before I Die” responses from both courses were extracted and de-identified. 

The 633 participants who responded to the “Before I Die” activity (20.31% of all Dying2learn enrolees) were predominantly female (93.3%), with a mean age of 49.75 (*SD* = 11.91, range of 19 to 81 years, with 22.2% aged 60+). Most participants resided in Australia (87.5%), with the remainder representing 16 other countries (predominantly English-speaking). The sample were generally well educated, with 67% having completed university studies, and 73.8% identifying themselves as having a health professional occupational background. 

### 2.2. Data Extraction and Analytical Approach

In qualitative methodology, content analysis sees the creation of “labels (codes) that can be applied to data in order to develop data into meaningful categories to be analysed and interpreted” [10] (p. 16). Open coding has been considered similar to brainstorming, with axial coding [11] a way of linking the concepts identified through this process [12]. We were guided by the principles of axial coding (drawn from grounded theory methodology) in that as we were coding we searched for repetitions of codes (which were merged) [10] and identified subthemes or in our case “descriptors”. We used the axial coding technique rather than adopting grounded theory in its entirety [12], with Kendall [13] clarifying that axial coding will not necessarily generate theory. According to Noble and Mitchell [14] grounded theory methodology would include simultaneous data collection and analysis (when in fact ours was retrospective), and to uncover social processes, which was not our intent. We developed codes from the data, however it was clear a priori, due to the nature of the exercise, what was likely to emerge from the data. We considered the bucket list approach to coding described by Periyakoil and colleagues [6] however using applied-coding methods (only applying their bucket list categories/schema to our data) would not be appropriate because it would not capture the more personalized nature of many of the responses we received.

All authors (D.R., L.M.-L., J.T.) independently coded the first 50 ‘Before I Die’ statements from both the 2016 and 2017 datasets. The 2016 dataset was the first time this activity was offered, and participants could include more than one sentiment—and often many more. This required coding of each component of the sentences and paragraphs detailing what they wanted to do before they die. Therefore, each participant’s statement could be assigned multiple coding labels. The second round of coding saw two authors (D.R., L.M.-L.) compiling the coding schemes independently derived by the three authors in the first round of coding. These themes were refined, examined for inter-relationships between the characteristics labelled, and then combined into one set of primary coding themes. The two authors (D.R., L.M.-L.) then returned to the 100 statements, independently coding them against the combined list of themes. Next, the coders compared their assignment of codes, and 100 percent exact agreement on the assignment of codes was initially reached for 49/100 statements. Two sub-theme codes under the category of family were identified as the source of major discrepancy (inward facing vs outward facing). Discussion and cross-verification determined that almost all responses fitted under both sub-themes, therefore making the sub-categories redundant. The re-allocation of the two sub-themes back into their major theme improved the 100 percent exact agreement to 64/100 statements. Overall, a total of 240 codes were assigned to the 100 statements and the independent coders matched on 190 of the 240 (79.2%) (114/141 for the 2016 cohort and 76/99 for the 2017 cohort). All remaining discrepancies in codes assigned to statements were reviewed and discussed until full consensus was reached. One more iteration of coming together a final time was performed to ensure cross-coding was successful and that the themes were clear, and a coding-assignment guide was developed. 

After the 100 statements were successfully coded, an additional 10 new (unseen) statements were then independently coded by two authors (D.R., L.M.-L.) for validation purposes, using the coding guide developed from the development cohort. No new coding themes emerged, suggesting theme saturation had been reached. One hundred percent agreement on the assignment of codes was reached for 8/10 statements. Overall, 28 out of the 30 codes assigned were identical, indicating an inter-rater agreement rate of 93.33%. The two discrepancies were discussed, resolved and amendments made to the coding-guide to assist with future coding. 

Two university psychology students independently coded all 633 responses to the ‘Before I Die’ activity for the presence of the individual themes pre-determined through qualitative analysis of the development cohort, based on the coding-guide created from coding the development cohort. Once all statements had been coded, the coders then compared their assignment of codes for inter-rater reliability. The two independent raters obtained 95.24% agreement on all the judgements made. Cohen’s Kappa was calculated to determine the proportion of inter-rater agreement over and above what would be expected from chance alone. The Cohen’s Kappa for inter-rater agreement was 0.789, *p* < 0.0005. This indicated a highly substantial level of agreement between the two independent raters [15]. All discrepancies in the codes were reviewed by both coders with an author (L.M.-L., D.R.), and discussed until consensus was reached. Any statements where difficulty was experienced in assigning themes were noted and discussed as a group, with authors DR and LML then determining if the addition of new themes was required. It was decided that religion/spirituality emerged as an additional theme due to mentions of God, spirituality and church in the full dataset. 

## 3. Results

Overall, the 633 statements by participants were assigned 1313 codes, or an average of 2.07 codes per statement. Twelve themes emerged from the data (Table 1). In order of frequency they were: family; do an activity; personal aspiration/self-identity; live life fully, happiness; love; wider social impact/greater good; peace; legacy; feel appreciative/gratitude; religion/spirituality; and health. 

A series of Chi-Square Tests of Independence were conducted to determine if there were gender or age-group differences in the themes present in people’s “Before I Die” statements. Statistically significant gender differences were only found for two themes. There was a significant association between a person’s statement mentioning “legacy” and gender *χ*^2^ (*df* = 1, *n* = 628) = 7.86, *p* = 0.005, with females less likely to mention legacy in their response to the “Before I Die” statement (4.4% vs. 14.3%). There was a significant association between a person’s statement mentioning ‘family’ and gender *χ*^2^ (*df* = 1, *n* = 628) = 5.28, *p* = 0.022, with females more likely to mention family in their “Before I Die” statement (39.2% vs. 21.4%). Next we examined differences between the three age categories of under 40, 40–59, and 60+ years. Only one statistically significant difference was found for age, with an association between a person’s statement mentioning “health” and age group *χ*^2^ (*df* = 2, *n* = 632) = 11.39, *p* = 0.003, with those in the 60+ age category being more likely to mention “health” in their response to “Before I Die” (5.7% vs. 1.7% for 40–59 age group and 0% for the under 40 age group). No other statistically significant differences were found. 

From the coding and analysis we were able to determine that there were three higher order themes which we have categorised as:

- Self-focussed/about me/*inward facing*;
- Others focussed/the greater good/*outward facing*;
- Family/about me and about others/*both inward and outward facing*.

**Figure 1:**
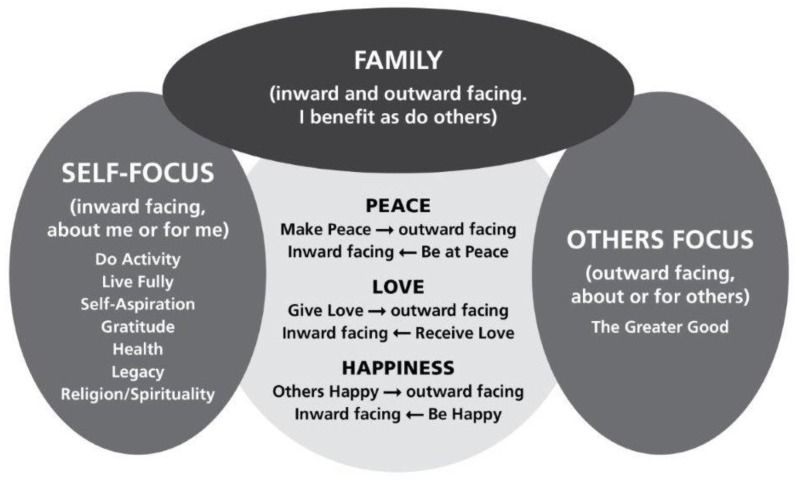
Visual representation of the generated themes.

We have used the inward and outward facing analogy above to describe how within themes there were sentiments that were able to be categorised as being either self-focussed or others focussed, or both. So for example: peace, love and happiness included comments such as “be loved” (inward facing, or about me, for me) and “love others” (outward facing, about or for others), “be at peace” (inward facing) or “make peace with someone” (outward facing), “be happy” (inward facing), and “for others to be happy” (outward facing). Overall, an “others focussed” theme was assigned 235 times, a “self-focussed” theme was assigned 837 times, and the theme relating to “family” was assigned 241 times. Figure 1 visually represents or interprets how we categorised participants’ responses relating to the themes and their meaning to the individual. The majority of responses were in relation to self, but even when there was tension between whether or not something was about me or about others, there was still an inward facing aspect.

## 4. Discussion

The constructivist “cMOOC” environment in Dying2Learn fostered an open, engaged community demonstrating a social connection reflected in their online posts and in subsequent conversations. There was a great sense of connectedness, support and kinship seen in the interaction in the MOOC participants as they discussed death and dying and made powerful disclosures of their own experiences. People often do not view things in light of their own mortality, but according to Rohrich [5] (p. 1129) “*Talking about death, considering death and planning for death is one of the healthiest ways to live*”. Another example is found in a public digital art installation that has facilitated socialisation in a community meeting place [7] translating “Before I Die” into another medium. As Candy Chang [1] says: “*At their greatest, our public spaces can nourish our well-being and help us see that we are not alone as we try to make sense of our lives*”. 

What we have seen in our cohort is how an assumed “well” community view what is important through the lens of death in a life context, and not in a health context. The top themes are: (1) family; (2) doing something; (3) self-aspiration; (4) living life fully; (5) happiness; and (6) love, all with over 100 mentions. Conversely, health receives only 14 mentions and is the lowest rated (12th theme), followed by religion at 15 mentions (11th). It is also important to also consider what has not been included. Statements mentioning materialistic factors such as finances were very rare, with only four mentions, related to mortgages or bills (which can also be seen as wanting to sort finances out for those who follow). 

From our results we can see that in our top five ‘family’ feature heavily in participant’s “Before I Die” responses. This includes both inward and outward facing aspects, and for many it was spending time with family, ensuring they will be ok or wanting to see children or grandchildren grow up. For example, in a wellness context, being well and considering family as important in our lives may mean taking off a Friday every now and again to spend time with them. In someone who is unwell, with family at the forefront of their minds and perhaps decision-making, then they may consider whether or not to have that extra round of chemotherapy, or how their medication will be managed at the end of life (e.g., whether or not to have sedation). 

Getting out and doing something was the second most popular theme and this included everything from bungee jumping to traveling, to finishing off things that had been started (e.g., a quilt). Establishing self-worth, self-identity, and personal aspirations was the third most popular theme and included things like “be the real me” or “find myself”, and alluded to personal journeys or insights not yet taken or discovered. Live life fully was fourth on the list, where participants want to live more fully when realising that life is finite. Fifth was “happiness”, either in terms of being happy (inward) or wanting to see others happy (outward). All of the five most common themes ultimately relate back to self (being happy, self-realisation), the unfinished things in life (go to Paris, live fully), the final words to be spoken (tell someone they are loved), or the upmost importance of family and friends and finding pleasure in life with them. 

We looked at our top five themes from 633 participants and compared them with those from Candy Chang’s book, “Before I Die” [1] and from the “bucket list” study [6]. See Table 2 below 

There are similarities in each of the top five from the three different sources, regardless of numbers, or country of origin. Travel features in all three (we coded this as an activity), as was family. It is interesting to note that there are also differences, for example, love which was rated second most popular on the “Before I Die” walls, and sixth most popular in the MOOC, but not mentioned at all in the “bucket list” exemplars. These differences will likely be due to the way in which people participated (what they wrote), and in the analysis of data, however, there is some level of commonality. The purpose of Candy Chang’s wall was to help us “grapple with death and meaning as a community today” (https://beforeidieproject.com/story/). This ethos of contemplating mortality has seen our participants articulate what it is they value in life, a reminder of all that is good, but also a reminder of what has not been achieved. We do need to consider that our “Before I Die” wall was offered virtually and importantly in a constructivist MOOC, a social environment that normalised talking about death and dying. The bucket list and the majority of “Before I Die” walls are often written as an individual response in a neutral context. 

Gilligan highlights different modes of thinking about relationships that are developed over time and include concern for our self (inward focus) concern for others (outward focus) and mutual concern (inward and outward facing) [16]. She views moral development through a feminist lens and deeply tied to relationships such as intimacy and care. In our study we found that females were significantly more likely to mention family in their “Before I Die” statements. Chentsova and Tsai [17] in addition to gender, highlight the importance of cultural and situational factors when looking at the self and emotion, in that culture can shape emotional reactions. We did not ask participants their cultural background but did however ask their age and unsurprisingly those in the 60+ age category were more likely to mention ‘health’ in their response to ‘Before I Die’ although the numbers are low.

While the concept of self-focus, focus on other people, and a mutual focus is not new in, for example, relationships theory [16,18], it has never been considered in relation to wishes at the end of life. From the “Before I Die” activity, themes in relation to what I want for myself, what I want to see for others and where I wish outcomes are of mutual benefit [19] were extrapolated from our data and realised a posteriori. We note the parallels between our categories determined using a theoretical methods and those presented in Gilligan’s [16] model of moral development. Perhaps our findings demonstrated similarities to Gilligan’s model due to our study sample being predominantly female. Future research with a more even gender distribution is needed to explore these issues. 

Periyakoil and colleagues [6] have used participants “bucket lists” as a clinical approach (discussion tool) in helping patients facing death to prioritise what is most important to them and to start important conversations. The “Before I Die” statement could be used in a similar context and is a way to ‘humanise’ these types of conversations. Clinicians need to consider that a person who is seriously ill is perhaps already thinking of these things and not necessarily about the next treatment option, Clinicians would be able to ascertain in these conversations what patients are thinking of as their long and short-term goals or wishes, and how realistic they are [6]. In reality though, the “Before I Die” movement is more in tandem with community Advance Care Planning, initiating and supporting conversations about death and dying, empowering people to think about things that are important while they are still alive and presumably, well. 

### 4.1. Strengths and Limitations

A strength within this study is that we do not often hear the voices of presumed healthy people (who aren’t receiving palliative care) and their wishes outside of a health-based context. This study is also arguably the first to systematically empirically code and analyse the way that people respond to the question “Before I Die, I want to…”. 

This study is limited in that our sample was a sample of convenience (of those self-selected into a MOOC on death and dying) and may not be representative of the general population due to this and the higher proportion of females and health professionals. It is possible our participants were more interested in this subject than the general population. There are also limitations in comparison between genders with so few male respondents. Information on participants’ health status was not collected, so while we assume the sample was a generally healthy cohort, this cannot be verified. What we have reported is an exploratory set of twelve themes and three higher order themes which could be influenced by the nature of the participants who chose to participate. There are also likely to be differences in those who chose not to participate in this activity. We also acknowledge that there could also have been an influence of perceived respectability and social desirability bias on the way people chose to respond. This may have impacted on the level of honesty in what people chose to write.

It is not clear how much time spent participating in the Dying2learn MOOC may have impacted on the way participants responded to this “Before I Die” activity at the end of the course, and their responses may have been different if it was completed at the beginning of the course. Despite this, the online course offered a unique opportunity to examine the views of the general public in an educational context instead of a health-related context. 

### 4.2. Future Directions

In the study reported here, the “Before I Die” scenario was considered within a non-health context with people presumed healthy. Future research could examine how people’s responses to ‘Before I Die’ might change when they are faced with the hypothetical scenario that they have only a short period of time to live. It’s possible that the themes in the responses given may be less ‘activity oriented’ in this circumstance and be more oriented towards what they really think matters the most. The themes identified in the current study tended to reflect different values people might hold, some with a focus on self and others with a focus on others. This ‘self’ and ‘others’ interpretation also needs further exploration as a legitimate and replicable direction.

Determining if people’s responses to questions about general life values are associated with the types of themes in their responses to the “Before I Die” activity is an important future research direction. It would also be valuable to determine if participant demographic characteristics are associated with responding to the “Before I Die” activity with different thematic responses. Future research could also investigate the impact of the “Before I Die”activity on participants’ death awareness and preparedness. For example, could participating in this activity increase the likelihood of a person subsequently having a discussion with family or health professional about end of life wishes? 

## 5. Conclusions

Reflecting on what is important and on what a person wishes to achieve or address before they die can be seen as a companion process to advance care planning which addresses what an individual wants to plan to manage their actual death. The “Before I Die” walls have commonly been used with the general population rather than populations who are unwell or indeed dying. In our population, the MOOC participants were again form the general community albeit the context was participation in a conversation about death and dying. We found that when asked to respond to “Before I die I want to…”, the most important themes were family, doing activities one has always wanted to do, fulfilling personal aspirations, living life fully, and happiness. 

This simple “Before I Die” activity encourages people to articulate values in their life more consciously, within the context of understanding that life is finite. It is hoped that this is a useful way for people to become more aware of their mortality and to live their life in a way that is congruent with what they consider to be what really matters the most to them in their lifetime. The potential of this activity for building death awareness and preparedness, and for understanding the impact on personal well-being are important considerations for future research. 

## Figures and Tables

**Table 1:** Themes identified.

	Theme	Frequency	Definition	Examples	Percent of Sample Who Mentioned Theme
1	“Family-related/relationships”	*n* = 241	Family, friends and relationships with loved ones. Inward and outward focussed sentiments were combined as while the focus can be on others, the person expressing the sentiment usually experiences something that will contribute to their own fulfilment (e.g., “I want to see my family do xxx”). It also includes the concept of having more time (e.g., “see children grow up”)	*“Before I die I would like the family re-united”; “know my loved ones will be alright”; “live to see my grandchildren”*	38.1%
2	“Do some activity”	*n* = 226	The desire to personally experience a specific activity, whether to be doing something adventurous, travelling or completing a particular task	*“write letters to my family and friends”; “Travel the world”; “Fly in an air balloon”*	35.7%
3	“Self-identity/personal aspirations”	*n* = 202	Personal goals people wished to achieve for themselves, such as find my calling, and where “self” is mentioned (self-worth)	*“I wish to be completely myself”; “I want to get to know myself a whole lot better”*	31.9%
4	“Live life fully”	*n* = 152	A desire to have no regrets, live in the moment, and enjoy life. Also when “live” is written on its” own (Live, Love, Laugh)	*“never look back in regret”; “Live with compassion and find meaning in every day”; “Live as if each day is my last”*	24.0%
5	“Happiness”	*n* = 113 I (*n* = 77) O (*n* = 23) B (*n* = 13)	A desire to be happy themselves or for others to be happy and encompassed mentions of “joy”, “enjoy”, “laugh”, and “smile”	*“Be happy*” (inward); “*See my children have happy, healthy, peaceful lives”* (outward)	17.9%
6	“Love”	*n* = 111 I (*n* = 21) O (*n* = 57) B (*n* = 33)	Love in many contexts, whether that be to receive love (inward facing) or give love to others (outward facing). Love is on its own assume it is inward facing	*“give love* (outward) *and receive love* (inward) *openly and with honesty”; “I want to experience unconditional love”* “*let all I know that I love them dearly” …* “*Share love and laughter with family and friends”*	17.5%
7	“Wider Social Impact/The Greater Good”	*n* = 88	Wishes to impact society more widely, by making the world a better place	*“help change the world”; “? Make this world a better place”; “Make a great contribution to humanity”*	13.9%
8	“ Peace”	*n* = 45 I (*n* = 24) O (*n* = 17) Both (*n* = 4)	Responses mentioned peace, and included both inward (be at peace themselves) and outward (make peace with others) facing aspects	*“Make peace with my ex husband”* (outward); *“be at peace with my life and choices I have made*” (inward)	7.1%
9	“Legacy”	*n* = 32	What they leave behind after death, such as “Make my mark”	*“Create a legacy of kindness and gratitude”; “Say that I have left some legacy no matter how small or how big”; “know that I mattered”*	5.1%
10	“Feel appreciative/Grateful/Gratitude”	*n* =24	Experiencing gratitude and appreciation	*“truly appreciate the value of life”; “I want to feel truly grateful for every day”*	3.8%
11	“Religion/Spirituality”	*n* = 15	Religious or spiritual element such as mentions of God, spirituality, faith, or church	*“Share the hope of Christ with others more!”; “feel safe and serene in the presence of God”; “grow in wisdom and love of God”*	2.4%
12	“Health”	*n* = 14	Health of self or family, such as “Be healthy” and, ‘“Pain free”	*“Remain healthy and independent”; “See my children grow into healthy, happy adults”; “Be painfree”*	2.2%

Note: Inward facing only (I), Outward facing only (O), and both inward and outward facing (B).

**Table 2:** Comparison of themes.

	Dying2Learn MOOC	Before I Die Walls	Bucket List
1	Family	Wellbeing	Travel
2	Activity	Love	Accomplish goal
3	Aspiration	travel	Milestone
4	Live fully	Helping	Family
5	Happiness	Family	Financial stability
